# Dietary choline intake and colorectal cancer: a cross-sectional study of 2005–2018 NHANES cycles

**DOI:** 10.3389/fnut.2024.1352535

**Published:** 2024-06-03

**Authors:** Xijuan Xu, Hongan Ying, Lili Huang, Weiwen Hong, Wenbin Chen

**Affiliations:** ^1^Department of Colorectal Surgery, The First Affiliated Hospital, Zhejiang University School of Medicine, Hangzhou, Zhejiang, China; ^2^Department of Anus and Intestine Surgery, Taizhou First People's Hospital, Taizhou, Zhejiang, China; ^3^Department of Geriatrics, Taizhou First People's Hospital, Taizhou, Zhejiang, China; ^4^Department of Emergency, Taizhou First People's Hospital, Taizhou, Zhejiang, China; ^5^Department of Anus and Intestine Surgery, Huangyan Hospital, Wenzhou Medical University, Taizhou, Zhejiang, China

**Keywords:** choline, colorectal cancer, dietary, NHANES, hypertension

## Abstract

**Background:**

It remains unclear if choline intake is associated with colorectal cancer. Therefore, we examined data from the National Health and Nutrition Examination Survey (NHANES).

**Methods:**

This cross-sectional study included 32,222 U.S. adults in the 2005–2018 NHANE cycles, among whom 227 reported colorectal cancer. Dietary choline was derived from 24-h recalls. Logistic regression estimated odds of colorectal cancer across increasing intake levels, adjusting for potential confounders.

**Results:**

After adjusting for sociodemographic variables, BMI, alcohol use, smoking status, comorbidities, and dietary factors (energy, fat, fiber, and cholesterol), the odds ratio (OR) for colorectal cancer was 0.86 (95% CI: 0.69–1.06, *p* = 0.162) per 100 mg higher choline intake. Across increasing quartiles of choline intake, a non-significant inverse trend was observed (Q4 vs. Q1 OR: 0.76, 95%CI: 0.37 ~ 1.55, *P*-trend = 0.23). Subgroup analyses revealed largely consistent associations, with a significant interaction by hypertension status (*P*-interaction =0.022).

**Conclusion:**

In this large, nationally representative sample of U.S. adults, higher dietary choline intake was not significantly associated with colorectal cancer odds after adjusting for potential confounders. However, a non-significant inverse trend was observed. Further prospective studies are needed to confirm these findings and elucidate the underlying mechanisms.

## Introduction

Colorectal cancer imposes a substantial global health burden, with over 1.9 million new cases and 935,000 deaths in 2020 (1). In the United States, colorectal cancer is the third most common cancer type and the second leading cause of cancer-related deaths (2). The high incidence, mortality, and associated healthcare costs underscore the importance of identifying effective preventive strategies, including modifiable dietary factors (3, 4).

Diet is considered one of the most important modifiable risk factors for colorectal cancer (5, 6). Previous studies have investigated the associations between various dietary components and colorectal cancer risk, including red and processed meat, fiber, and certain micronutrients (7). However, the evidence for many of these relationships remains inconsistent or inconclusive, partly due to differences in study designs, populations, and methods of dietary assessment (8). Therefore, it is important to continue exploring the potential roles of other dietary factors, such as choline, in colorectal carcinogenesis.

Choline is an essential nutrient involved in various biological processes, such as maintaining cell membrane integrity, methylation reactions, and neurotransmitter synthesis (9). Some experimental studies suggest that choline deficiency may influence carcinogenesis by modulating cell proliferation, apoptosis, and signal transduction pathways (10, 11). However, the evidence from human studies remains limited and inconsistent (12). Moreover, the potential mechanisms linking dietary choline intake to colorectal cancer risk in humans are not fully understood and warrant further investigation.

To date, only a few epidemiological studies have directly investigated the relationship between dietary choline intake and colorectal cancer risk, with inconsistent results. A case–control study in China found an inverse association between choline intake and colorectal cancer risk (13), while another case–control study in Iran reported a positive association (14). However, these studies were limited by their relatively small sample sizes and potential residual confounding. A prospective cohort study in the United States found no significant association between choline intake and colorectal cancer risk in men (15), but it did not include women or examine potential effect modifiers. Given these limitations and inconsistencies, further large-scale studies are needed to clarify the relationship between dietary choline and colorectal cancer risk in diverse populations.

To address the limitations and inconsistencies in previous studies, we aimed to investigate the relationship between dietary choline intake and colorectal cancer risk in a large, nationally representative sample of U.S. adults. We also sought to explore potential effect modification by several factors, such as age, sex, and comorbidities. By using data from the National Health and Nutrition Examination Survey (NHANES), which includes detailed dietary assessments and comprehensive covariate information, we sought to provide more definitive evidence on this relationship while adjusting for important confounders. Understanding the role of dietary choline in colorectal cancer risk may help inform future dietary recommendations and prevention strategies.

Elucidating the relationship between dietary choline and colorectal cancer risk may contribute to a better understanding of the complex role of diet in cancer prevention. Although our study cannot directly inform dietary guidelines or prevention strategies, it may provide valuable insights for future research on the potential mechanisms linking choline metabolism to colorectal carcinogenesis.

## Methods

### Data sources and population

This cross-sectional study analyzed data from the 2005–2018 cycles of the NHANES. The NHANES is a program of the National Center for Health Statistics that uses stratified, multistage probability sampling to obtain nationally representative samples of the United States noninstitutionalized population (16). The current study linked NHANES datasets from the dietary interview, questionnaire, and household interview components (17).

**Figure 1:**
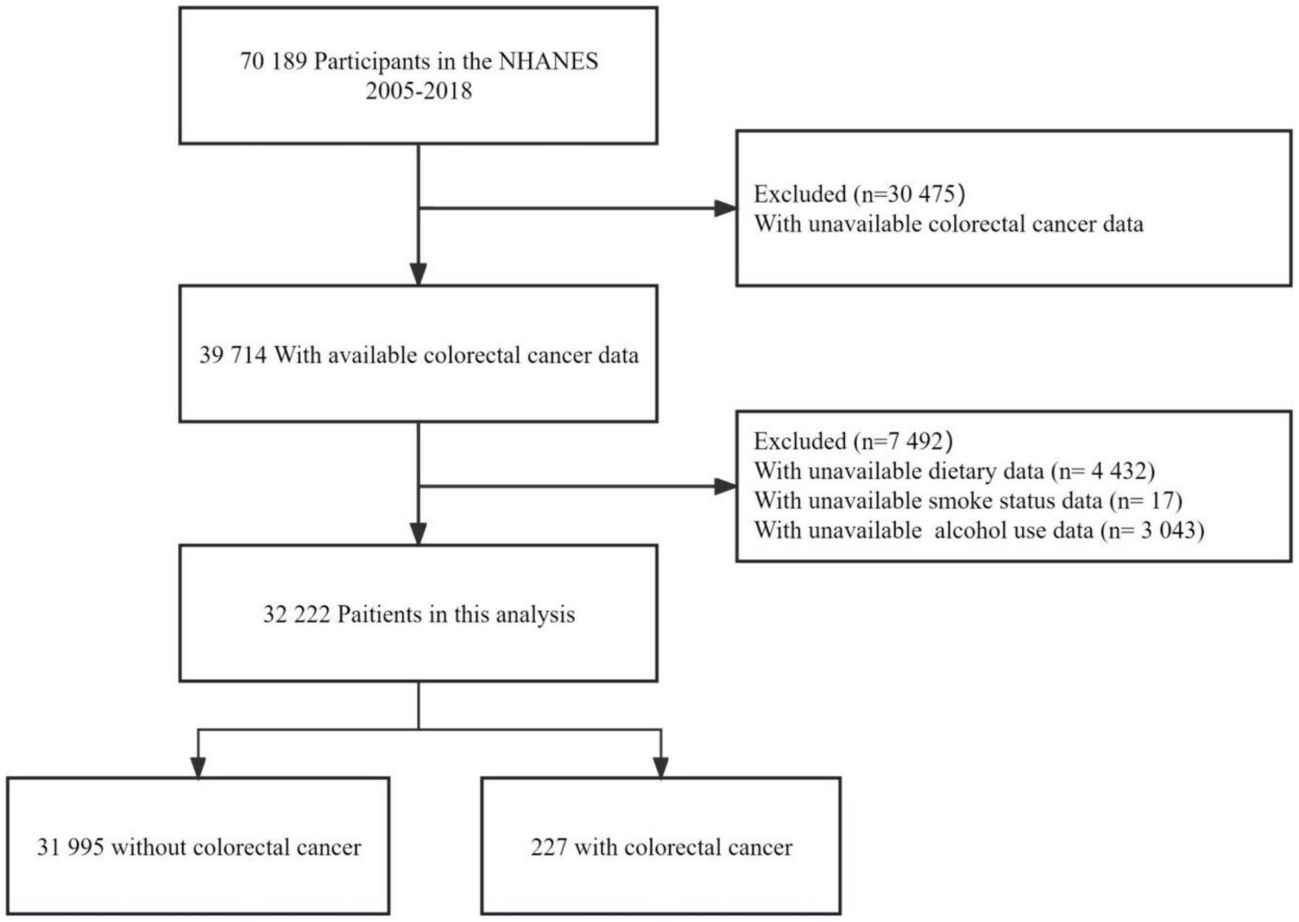
The flow chart of the study.

The full NHANES 2005–2018 sample consisted of 70,189 participants. Participants were excluded from this analysis if they did not have available data on colorectal cancer status, dietary intake, smoking status, or alcohol use, resulting in an eligible analytic sample of 32,222 adults (aged 20 years and older). Of these, 227 individuals (0.7%) reported being previously diagnosed with colorectal cancer. Details on exclusion criteria are provided in Figure 1. The study population was distributed across all age, gender, race/ethnicity, income, and education groups. Due to the low percentage of missing data (varying from 0 to 1.9%), no imputation was carried out.

### Colorectal cancer definition

Colorectal cancer status was determined using the medical conditions files from the NHANES surveys, where participants were asked “Has a doctor or other health care professional ever told {you/SP} that {you/s/he} had cancer or a malignancy of any kind?” If yes, they were asked to specify the kind of cancer. Interviewers coded the cancer types reported, which were subsequently aggregated into categories (18).

Colorectal cancer cases included participants who reported being told by a doctor or healthcare professional that they had either colon, rectal or colorectal cancer specifically. Those who reported other gastrointestinal malignancies were excluded. Separate sensitivity analyses were conducted for colon and rectal cancer subgroups coded analogously (19). Cancer mortality was additionally determined through probabilistic linkage with death certificate National Death Index records (20).

### Dietary assessment

Dietary choline intake was derived from the two in-person 24-h dietary recalls administered by trained staff using the United States Department of Agriculture (USDA) Automated Multiple-Pass Method (21). Choline content of all reported food and beverages consumed by participants over the two recall days were determined using the USDA Food and Nutrient Database for Dietary Studies and averaged to calculate daily total choline intake from foods. Choline intake was estimated by averaging values from the two recalls. If only one recall was available, that intake was used (22).

Supplemental choline intake was derived from the two 24-h dietary recalls, where participants self-reported types and amounts of all dietary supplements consumed. Total choline from supplements was averaged over the two recall days together with dietary choline to calculate total choline intake. Separate analyses were conducted for dietary choline and total choline from both foods and supplements over identical 24-h recall periods.

### Covariates

Based on prior literature, analyses adjusted for the following potential confounding factors: age, sex, race/ethnicity, educational level, marital status, family income, body mass index (BMI), smoking status, alcohol intake status, presence of diabetes, hypertension diagnosis, hyperlipidemia diagnosis, history of cardiovascular disease (CVD), diagnosis of chronic obstructive pulmonary disease (COPD), level of physical activity in metabolic equivalents (METs), total dietary energy intake, and dietary fat intake.

Race and ethnicity groups were categorized as: Mexican American, non-Hispanic Asian, non-Hispanic Black, non-Hispanic White, other Hispanic, and other/multiracial. Educational attainment was divided into 3 levels: high school or less, some college, and college graduate or higher. Family income was trichotomized based on the family poverty income ratio into low income (≤1.5), medium income (>1.5 to 3.5), and high income (>3.5). Marital status had 4 categories: married, never married, living with partner, and other (widowed, divorced, separated).

Smoking status had 3 groups: never smoked, former smoker (quit after ≥100 cigarettes), and current smoker. Alcohol intake status was determined by the survey question on having ≥12 alcoholic drinks in a given year, with those answering “yes” defined as drinkers. Hypertension: Defined as systolic blood pressure ≥ 130 mmHg, diastolic blood pressure ≥ 80 mmHg, self-reported prior diagnosis of hypertension, or reported antihypertensive medication use. Diabetes was defined as using antidiabetic medication or having a fasting glucose ≥126 mg/dL. Metabolic syndrome was classified using Adult Treatment Panel III criteria. CVD: Defined as having coronary heart disease, angina, myocardial infarction, stroke, or congestive heart failure based on self-reported physician diagnoses. COPD: Based on affirmative responses to survey questions regarding chronic bronchitis or emphysema diagnoses. Physical activity: Physical activity was expressed as metabolic equivalents (METs) per week, calculated from self-reported frequencies and durations of moderate and vigorous intensity leisure-time activities multiplied by the corresponding MET value for each activity. Dietary energy intake: Total caloric intake in kilocalories per day derived from the nutrient profiles of all foods and beverages reported on 24-h dietary recalls using the USDA Food and Nutrient Database. Dietary fat intake: Total fat intake in grams per day derived from the nutrient profiles of all foods and beverages reported on 24-h dietary recalls using the USDA Food and Nutrient Database (23).

### Statistical analysis

Participant characteristics were summarized using means ± standard errors or frequencies and weighted percentages as appropriate. Univariable comparisons by colorectal cancer status were tested using chi-square tests and survey-weighted linear regressions.

The association between dietary choline intake, modeled as both a continuous and categorical quartile variable, and colorectal cancer odds was analyzed using survey-weighted logistic regression. Three hierarchical models were constructed with progressive adjustment for potential confounders. Model 1 adjusted for sociodemographic characteristics including age, sex, race/ethnicity, education, family income, and marital status. Model 2 additionally adjusted for health behaviors and comorbidities including BMI, smoking, alcohol intake, hypertension, diabetes, cardiovascular disease, and chronic pulmonary disease. Model 3 further adjusted for dietary factors including energy, fat, fiber and cholesterol intake. Non-linear relationships were evaluated using restricted cubic spline modeling with four knots placed at the 5th, 35th, 65th, and 95th percentiles of the dietary choline intake distribution. The restricted cubic spline analyses did not account for the complex sampling design of NHANES.

Subgroup analyses and interaction testing assessed whether associations differed across age, sex, hypertension, diabetes, alcohol user and smoke status. Sensitivity analyses evaluated the robustness of findings under different assumptions including multiple outcome definitions, use of propensity score matching, and other alternate specifications detailed in Table 3. Propensity score matching was performed as a sensitivity analysis to compare participants with high versus low dietary choline intake. The propensity score was estimated using a logistic regression model that included the following covariates: age, sex, race/ethnicity, education, family income, marital status, BMI, smoking status, alcohol use, and comorbidities (hypertension, diabetes, hyperlipidemia, cardiovascular disease, and chronic obstructive pulmonary disease). Participants were then matched 1:1 based on their propensity scores using the nearest neighbor method with a caliper width of 0.2 times the standard deviation of the logit of the propensity score. All analyses applied NHANES survey weights and accounted for the complex sampling design. Results were considered statistically significant at a two-sided alpha level of 0.05 unless otherwise specified. All analyses were conducted in R version 4.2.2.

## Results

### Characteristics of participants

The final analytic sample consisted of 32,222 participants, of which 227 (0.7%) reported a previous diagnosis of colorectal cancer (Table 1). Those with colorectal cancer were older on average (mean age 67.1 vs. 47.2 years, *p* < 0.001) and had a higher proportion of females (58.2% vs. 51.4%, *p* = 0.12) than those without colorectal cancer. Compared to participants without colorectal cancer, a higher proportion of those with colorectal cancer were non-Hispanic white (82.4% vs. 67.9%) and a lower proportion were Mexican American (1.8% vs. 8.5%) or Non-Hispanic black (8.7% vs. 11.1%) (*p* = 0.003 for differences across groups). Participants with colorectal cancer were also more likely to be widowed, divorced or separated (41.8% vs. 18.6%, *p* < 0.001). No significant differences were observed in mean BMI or the distribution of family income levels or education categories between those with and without colorectal cancer.

**Table 1:** Characteristics of participants in the NHANES 2005–2018 cycles.

Characteristic	Participants^a^	Without colorectal cancer	With colorectal cancer	*p* value
	Total (*n* = 32,222)	(*n* = 31,995)	(*n* = 227)	
Age (mean ± se)	47.33 ± 0.25	47.21 ± 0.25	67.11 ± 1.01	< 0.001
Sex (mean ± se)				0.12
Female	16,392 (51.43)	16,277 (51.39)	115 (58.17)	
Male	15,830 (48.57)	15,718 (48.61)	112 (41.83)	
Race and ethnicity^b^				0.003
Mexican American	5,058 (8.44)	5,049 (8.48)	9 (1.78)	
Non-Hispanic Black	6,886 (11.10)	6,835 (11.11)	51 (8.73)	
Non-Hispanic White	14,017 (67.95)	13,874 (67.86)	143 (82.38)	
Other Hispanic	3,036 (5.24)	3,023 (5.25)	13 (2.82)	
Other Race^c^	3,225 (7.28)	3,214 (7.30)	11 (4.29)	
Marital status				< 0.001
Living with partner	2,687 (8.26)	2,684 (8.30)	3 (1.23)	
Married	16,570 (54.12)	16,456 (54.13)	114 (52.92)	
Never married	5,869 (18.88)	5,856 (18.96)	13 (4.01)	
Other^d^	7,096 (18.75)	6,999 (18.61)	97 (41.84)	
Family income^e^				0.28
High	16,270 (61.30)	16,174 (63.51)	96 (57.85)	
Low	4,833 (11.03)	4,793 (11.42)	40 (12.01)	
Medium	9,689 (24.25)	9,608 (25.08)	81 (30.14)	
Education				0.84
College graduate or above	7,424 (29.27)	7,382 (29.29)	42 (25.91)	
High school or less	15,186 (38.68)	15,066 (38.68)	120 (39.45)	
Some college	9,589 (32.01)	9,524 (31.99)	65 (34.63)	
BMI	29.04 ± 0.09	29.04 ± 0.09	29.18 ± 0.48	0.78
Alcohol drinker^f^				< 0.001
No	9,976 (24.44)	9,866 (24.33)	110 (42.40)	
Yes	22,246 (75.56)	22,129 (75.67)	117 (57.60)	
Smoke status				< 0.001
Former	7,852 (24.68)	7,756 (24.57)	96 (44.05)	
Never	17,741 (54.93)	17,643 (54.99)	98 (43.34)	
Now	6,629 (20.39)	6,596 (20.44)	33 (12.61)	
Hypertension				< 0.001
No	18,576 (62.00)	18,524 (62.21)	52 (27.62)	
Yes	13,643 (37.99)	13,468 (37.79)	175 (72.38)	
Diabetes				< 0.001
No	25,666 (84.94)	25,526 (86.12)	140 (64.22)	
Yes	5,942 (13.83)	5,855 (13.88)	87 (35.78)	
Hyperlipidemia				0.01
No	9,839 (31.01)	9,789 (31.08)	50 (20.01)	
Yes	22,382 (68.99)	22,205 (68.92)	177 (79.99)	
CVD				< 0.001
No	28,695 (91.33)	28,538 (91.45)	157 (72.25)	
Yes	3,524 (8.66)	3,454 (8.55)	70 (27.75)	
COPD				< 0.001
No	30,711 (95.53)	30,512 (95.58)	199 (87.46)	
Yes	1,510 (4.47)	1,482 (4.42)	28 (12.54)	
Physical activity (MET) (mean ± se)	4312.25 ± 80.38	4322.15 ± 80.97	2224.49 ± 417.34	< 0.001
Dietary energy (kcal/d) (mean ± se)	2156.16 ± 8.70	2158.18 ± 8.76	1815.59 ± 61.31	< 0.001
Dietary fat (g/d) (mean ± se)	83.41 ± 0.45	83.48 ± 0.45	70.33 ± 2.72	< 0.001
Dietary fiber (g/d) (mean ± se)	16.95 ± 0.14	16.95 ± 0.14	15.54 ± 0.70	0.04
Dietary choline (mg/d) (mean ± se)	336.55 ± 1.72	336.86 ± 1.74	284.17 ± 11.57	< 0.001

NHANES, National Health and Nutrition Examination Survey; CVD, cardiovascular disease; COPD, chronic obstructive pulmonary disease; BMI, body mass index; MET, total metabolic equivalent. ^a^Data are presented as unweighted number (weighted percentage or weighted mean ± se) unless otherwise indicated. ^b^Race and ethnicity were self-reported. ^c^Included multiracial participants. NHANES did not provide a detailed list of all races and ethnicities included in this category. ^d^Included widowed, divorced, or separated individuals. ^e^Categorized into the following 3 levels based on the family poverty income ratio: low income (1.5), medium income (>1.5 to 3.5), and high income (>3.5). ^f^Determined by answering the following question: “In any 1 year, have you had at least 12 drinks of any type of alcoholic beverage?”.

Those with colorectal cancer were more likely to abstain from alcohol (42.4% vs. 24.3%), be former smokers (44.0% vs. 24.6%), have hypertension (72.4% vs. 37.8%), diabetes (35.8% vs. 13.9%), hyperlipidemia (80.0% vs. 69.0%), cardiovascular disease (27.7% vs. 8.5%), and chronic obstructive pulmonary disease (12.5% vs. 4.4%) (all *p* < 0.001). They also had lower levels of physical activity (2,224 vs. 4,322 METs, *p* < 0.001) and lower dietary intake of total energy (1815 vs. 2,158 kcal/day), fat (70.3 vs. 83.5 g/day) and choline (284 vs. 337 mg/day) (all *p* < 0.001).

### Association between dietary choline and colorectal cancer

When modeled as a continuous variable, higher dietary choline intake was associated with lower odds of having colorectal cancer in the crude model (Model 2) (OR per 100 mg/d: 0.85, 95% CI: 0.79–0.93, *p* < 0.001) (Table 2). This association remained significant after adjusting for sociodemographic variables (Model 1, OR: 0.91, 95% CI: 0.83–1.00, *p* = 0.043). Further adjustment for comorbidities and health behaviors (Model 2) did not substantially change the results (OR: 0.90, 95% CI: 0.82–0.98, *p* = 0.023). Additional adjustment for dietary factors including energy, fat, fiber and cholesterol intake did not significantly affect the results (Model 3, OR: 0.86, 95% CI: 0.69–1.06, *p* = 0.162).

**Table 2:** Association of dietary choline with colorectal cancer among participants in the NHANES 2005–2018 cycles.

Dietary Choline (Cases/participants)	100 mg/d (mean ± se)	Crude Model OR (95%CI)	*P*	Model 1 OR (95%CI)	*P*	Model 2 OR (95%CI)	*P*	Model 3 OR (95%CI)	*P*
Continuous variable (227/32222)	3.37 ± 0.017	0.85 (0.79 ~ 0.93)	<0.001	0.91 (0.83 ~ 1.00)	0.043	0.9 (0.82 ~ 0.98)	0.023	0.86 (0.69 ~ 1.06)	0.162
Quartile									
Q1 (75/7976)	1.37 ± 0.005	Reference		Reference		Reference		Reference	
Q2 (65/8061)	2.40 ± 0.003	0.86 (0.61 ~ 1.20)	0.362	0.86 (0.61 ~ 1.21)	0.375	0.82 (0.58 ~ 1.16)	0.260	0.86 (0.59 ~ 1.26)	0.442
Q3 (45/8072)	3.50 ± 0.004	0.59 (0.41 ~ 0.86)	0.005	0.64 (0.43 ~ 0.94)	0.022	0.64 (0.43 ~ 0.95)	0.026	0.70 (0.43 ~ 1.14)	0.149
Q4 (42/8113)	5.44 ± 0.021	0.55 (0.38 ~ 0.80)	0.002	0.74 (0.49 ~ 1.11)	0.143	0.66 (0.43 ~ 1.01)	0.056	0.76 (0.37 ~ 1.55)	0.447
*P* for Trend		<0.001		0.046		0.020		0.228	

Model 1, Adjusted for sociodemographic variables (age, sex, marital status, race and ethnicity, education, family income). Model 2, Adjusted for sociodemographic variables, BMI, alcohol use, smoking status and comorbidities (hypertension, diabetes, hyperlipidemia, CVD, COPD). Model 3, Adjusted for sociodemographic variables, BMI, alcohol use, smoking status, comorbidities, dietary factors (energy, fat, fiber and cholesterol). NHANES, National Health and Nutrition Examination Survey; CVD, cardiovascular disease; COPD, chronic obstructive pulmonary disease; BMI, body mass index.

When participants were categorized into quartiles based on their dietary choline intake, those in the second (OR: 0.86, 95% CI: 0.59–1.26, *p* = 0.442), third (OR: 0.70, 95% CI: 0.43–1.14, *p* = 0.149), and fourth quartiles (OR: 0.76, 95% CI: 0.37–1.55, *p* = 0.447) had lower odds of prevalent colorectal cancer compared to those in the lowest quartile (Q1) after adjusting for sociodemographic variables, BMI, alcohol use, smoking status, comorbidities, and dietary factors (energy, fat, fiber and cholesterol) in Model 3. However, these associations were not statistically significant, and the trend across quartiles was attenuated (*P*-trend = 0.228).

### Restricted cubic splines for dietary choline intake and colorectal cancer

**Figure 2:**
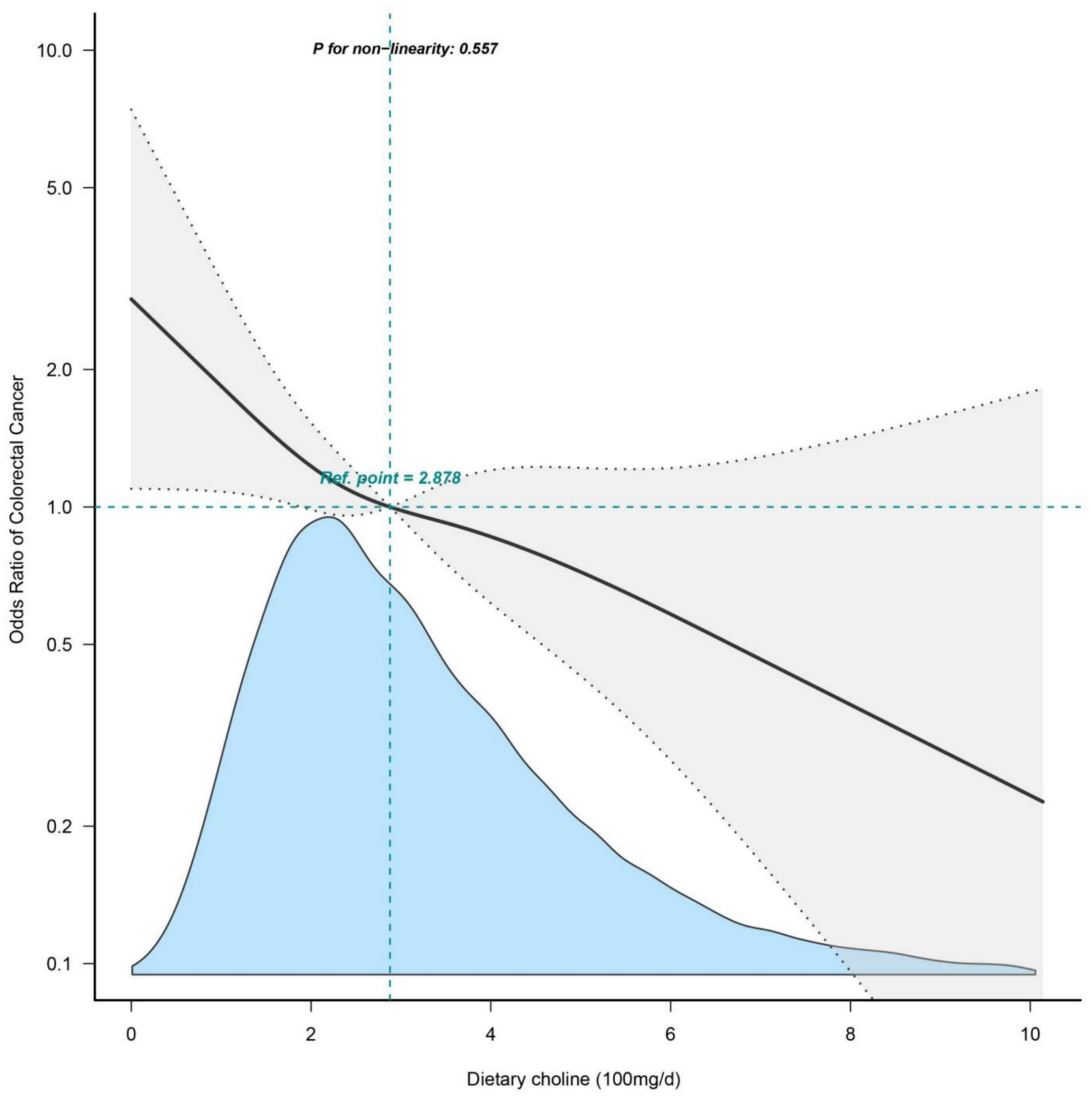
Restricted cubic splines of dietary choline intake and colorectal cancer. Adjusted for sociodemographic variables, BMI, alcohol use, smoking status and comorbidities (hypertension, diabetes, hyperlipidemia, CVD, COPD). NHANES, National Health and Nutrition Examination Survey; CVD, cardiovascular disease; COPD, chronic obstructive pulmonary disease; BMI, body mass index.

The association between dietary choline intake and the odds of prevalent colorectal cancer was further explored using restricted cubic splines (Figure 2). The odds ratio curve showed a gradual decrease but remained largely linear even at higher intake levels. The test for non-linearity was not statistically significant (*p* = 0.557), confirming there was no significant departure from a linear relationship over the full range of choline intake.

### Subgroup analyses

**Figure 3:**
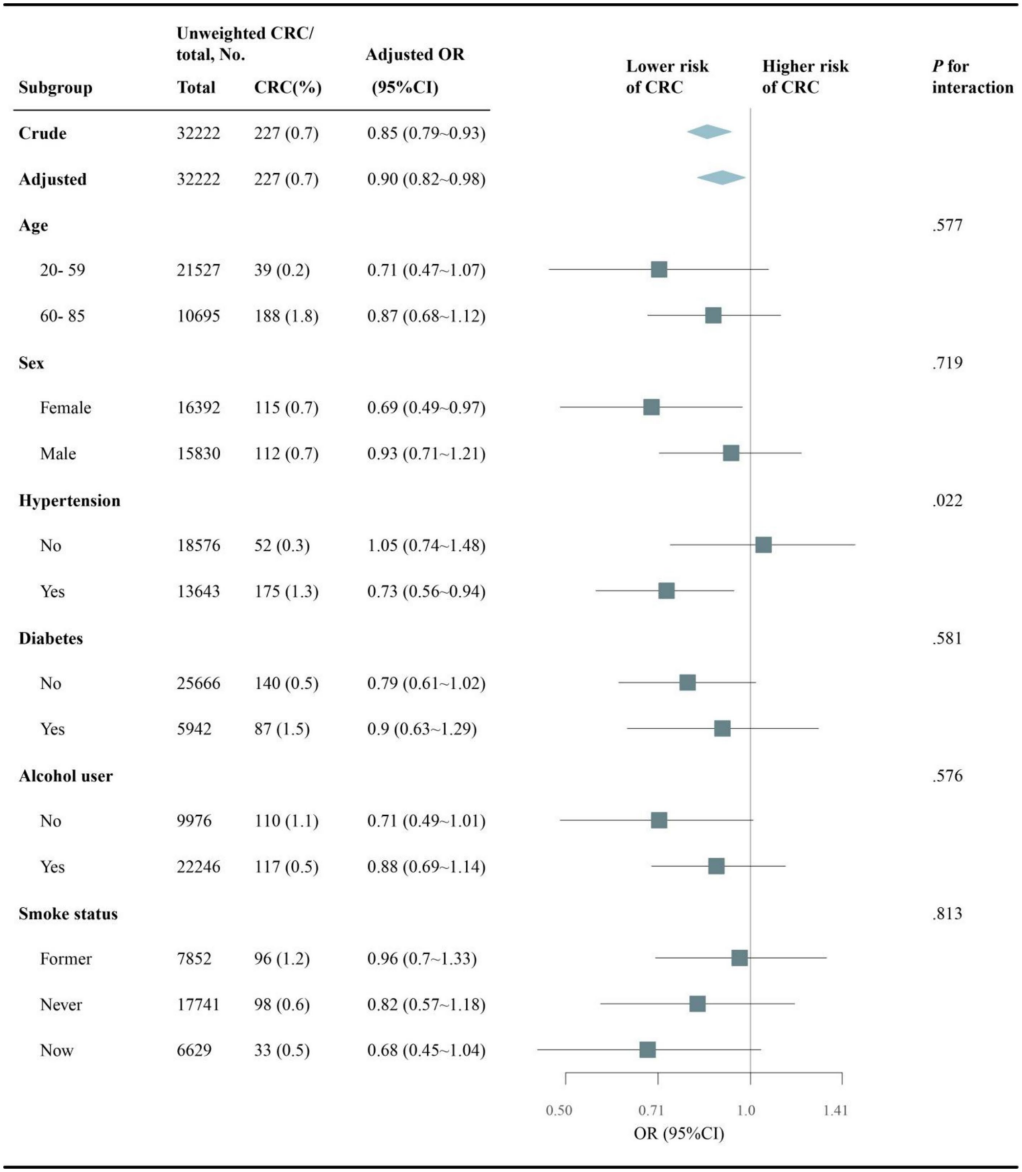
Subgroup analysis of choline intake and CRC risk stratified by baseline characteristics. Adjusted for sociodemographic variables, BMI, alcohol use, smoking status and comorbidities (hypertension, diabetes, hyperlipidemia, CVD, COPD). NHANES, National Health and Nutrition Examination Survey; CVD, cardiovascular disease; COPD, chronic obstructive pulmonary disease; BMI, body mass index.

Subgroup analyses evaluated whether the association between dietary choline intake and colorectal cancer differed across subgroups (Figure 3). Overall, higher dietary choline intake was associated with lower colorectal cancer odds after adjustment (OR per 100 mg/d: 0.90, 95% CI: 0.82–0.98). The inverse association did not significantly differ by age, sex, diabetes status, alcohol intake or smoking status (all *p*-interaction >0.05). However, a significant interaction by hypertension status was observed (*p* = 0.022), where the association was stronger among those without hypertension (OR: 1.05, 95% CI: 0.74–1.48) versus those with hypertension (OR: 0.73, 95% CI: 0.56–0.94).

### Sensitivity analyses

Several sensitivity analyses were conducted to evaluate the robustness of the association between dietary choline intake and colorectal cancer (Table 3). When missing alcohol use and smoke data were accounted for using dummy variable coding, the inverse association persisted (adjusted OR per 100 mg/d: 0.90, 95% CI: 0.81–0.98, *p* = 0.022). Defining outcome alternatively as colon cancer cases also showed consistent results (adjusted OR: 0.89, 95% CI: 0.81–0.98, *p* = 0.019). However, the association was attenuated when limited to rectal cancer cases (adjusted OR: 0.90, 95% CI: 0.63–1.29, *p* = 0.572). When modeling colorectal cancer mortality as the outcome (adjusted OR: 0.83, 95% CI: 0.65–1.05, *p* = 0.122) and using total choline intake from diet and supplements as the exposure similarly showed an inverse trend (adjusted OR: 0.89, 95% CI: 0.79–1.00, *p* = 0.052). In propensity score matched analyses comparing participants with high versus low dietary choline intake, the high intake group had significantly lower colorectal cancer odds (OR: 0.64, 95% CI: 0.46–0.88, *p* = 0.007). The inverse choline-colorectal cancer association remained robust in multiple sensitivity analyses, supporting the primary study conclusions.

**Table 3:** Sensitivity analyses.

Analysis^a^	Colorectal cancer/total participants, No.	Adjusted OR (95% CI)	*P* value
Excluding only participants missing data on dietary choline and colorectal cancer (dummy variable coding for missing data)			
Dietary choline	297/39714	0.90 (0.81, 0.98)	0.022
Outcome definition as colon cancer			
Dietary choline	218/32222	0.89 (0.81, 0.98)	0.019
Outcome definition as rectal cancer			
Dietary choline	15/32222	0.9 (0.63, 1.29)	0.572
Outcome definition as colorectal cancer mortality			
Dietary choline	49/23831	0.83 (0.65, 1.05)	0.122
Exposure as dietary and supplement choline			
Total choline	147/19606	0.89 (0.79, 1.00)	0.052
Propensity score matching			
Dietary choline (low)	95/11075	Reference	
Dietary choline (high)	61/11075	0.64 (0.46, 0.88)	0.007

^a^Adjusted for sociodemographic variables, BMI, alcohol use, smoking status and comorbidities (hypertension, diabetes, hyperlipidemia, CVD, COPD). NHANES, National Health and Nutrition Examination Survey; CVD, cardiovascular disease; COPD, chronic obstructive pulmonary disease; BMI, body mass index.

## Discussion

In this large, nationally representative cross-sectional study of U.S. adults, we found that higher dietary choline intake was inversely associated with the odds of colorectal cancer in the age- and sex-adjusted model (Model I) and the model further adjusted for sociodemographic factors, lifestyle behaviors, and comorbidities (Model II). However, after additional adjustment for other dietary factors (Model III), the inverse association was attenuated and no longer statistically significant. This suggests that the potential association between dietary choline and colorectal cancer risk may be largely explained by confounding from other dietary factors that are associated with both choline intake and colorectal cancer risk. It is also important to note that the relatively small number of colorectal cancer cases (n = 227) in our study, coupled with the extensive adjustment for covariates in Model III, may have increased the possibility of over-adjustment or overfitting, which could have affected the precision and stability of the estimates. Therefore, our findings should be interpreted with caution, and further studies with larger sample sizes and careful consideration of potential confounders are warranted to confirm these results.

Our findings are inconsistent with some previous studies that have reported inverse or positive associations between dietary choline intake and colorectal cancer risk. For example, a case–control study in China found a significant inverse association (13), while another case–control study in Iran reported a significant positive association (14). However, these studies were limited by their relatively small sample sizes, potential selection bias, and inadequate adjustment for important confounders, such as other dietary factors. In contrast, a prospective cohort study in the United States found no significant association between choline intake and colorectal cancer risk among men (15), which is more consistent with our findings. The discrepancies between these studies may be attributed to differences in study designs, populations, and methods of exposure assessment. Moreover, the inconsistent results across studies highlight the complexity of the relationship between dietary choline and colorectal cancer risk and the potential influence of residual confounding by other dietary and lifestyle factors. Our study, with its nationally representative sample, comprehensive adjustment for potential confounders, and exploration of effect modification, adds valuable insights to this area of research.

Although our study did not find a significant association between dietary choline intake and colorectal cancer risk after adjusting for potential confounders, it is still important to discuss the potential biological mechanisms that have been suggested by experimental studies. Choline is an essential nutrient involved in various physiological processes, such as cell membrane synthesis, lipid metabolism, and DNA methylation (24). Some animal and *in vitro* studies have indicated that choline deficiency may lead to abnormal DNA methylation, increased cell proliferation, and altered cell signaling pathways, which could potentially contribute to carcinogenesis (11, 25). However, it is crucial to note that the evidence for these mechanisms in human populations is still limited and inconsistent. While some observational studies have reported associations between choline intake or blood levels and cancer risk (26), others have found no significant relationships (15). Moreover, the complex interplay between choline metabolism, gut microbiota, and other dietary factors in the context of colorectal cancer development remains to be elucidated (27). Therefore, further well-designed epidemiological studies, particularly prospective cohort studies with repeated measurements of choline intake and relevant biomarkers, are needed to clarify the potential role of choline in colorectal carcinogenesis and to explore the underlying mechanisms in human populations.

Our subgroup analyses revealed a significant interaction between choline intake and hypertension status, with a stronger inverse association between choline intake and colorectal cancer odds among those with hypertension compared to those without. While the exact mechanisms underlying this interaction are not fully understood, there are several potential explanations. Hypertension is associated with endothelial dysfunction, oxidative stress, and inflammation, which may contribute to an increased risk of colorectal cancer. Choline, as a precursor for the synthesis of phosphatidylcholine, plays a crucial role in maintaining cell membrane integrity and reducing inflammation (28, 29). Additionally, choline is involved in one-carbon metabolism, which regulates DNA methylation and gene expression. Abnormalities in one-carbon metabolism have been linked to both hypertension and colorectal cancer (30, 31). It is possible that individuals with hypertension may have a higher requirement for choline to counteract the adverse effects of oxidative stress and inflammation, leading to a more pronounced protective effect of higher choline intake against colorectal cancer. In our subgroup analyses, we also examined potential differences in the association between choline intake and colorectal cancer odds stratified by sex. While some previous studies have suggested that the relationship between dietary factors and colorectal cancer risk may differ between men and women (32, 33), we did not observe a statistically significant interaction by sex in our study. This suggests that the inverse association between choline intake and colorectal cancer odds was consistent across both men and women in this U.S. population.

Our study has several limitations that should be considered when interpreting the results. First, the cross-sectional design precludes the establishment of a temporal relationship between dietary choline intake and colorectal cancer risk, and reverse causation cannot be ruled out. It is possible that individuals with colorectal cancer may have altered their dietary habits, including choline intake, as a result of their diagnosis or treatment. Prospective cohort studies with repeated assessments of choline intake and long-term follow-up are needed to address this limitation. Second, the dietary data were based on self-reported 24-h dietary recalls, which may be subject to measurement errors, such as underreporting or misreporting of food intake. Although we averaged choline intake from two non-consecutive 24-h recalls to reduce the impact of day-to-day variability, this method may not fully capture long-term habitual intake. Third, despite adjusting for a wide range of potential confounders, we cannot rule out the possibility of residual confounding by unmeasured or incompletely measured factors, such as family history of colorectal cancer, genetic susceptibility, and betaine. Finally, the relatively small number of colorectal cancer cases in our study may have limited the statistical power to detect a significant association and the ability to conduct more detailed subgroup analyses. Future studies with larger sample sizes are warranted to confirm our findings and to further explore potential effect modifiers.

## Conclusion

In this large, nationally representative cross-sectional study of US adults, we found no significant association between dietary choline intake and colorectal cancer risk after adjusting for potential confounders. However, a non-significant inverse trend was observed. Although subgroup analyses suggested a potential interaction by hypertension status, this finding requires further investigation. Future prospective cohort studies with larger sample sizes and repeated assessments of choline intake are needed to confirm these results and explore the underlying mechanisms.

## Data availability statement

The datasets analyzed for this study are available via the CDC NHANES website (https://www.cdc.gov/nchs/nhanes).

## Ethics statement

The NHANES surveys obtained informed consent from all original participants at the time of data collection, and protect participant confidentiality by anonymizing records before public release. As this study involved analysis of existing de-identified data from the publicly available NHANES database, it was considered exempt from the institutional review board of the First People’s Hospital of Taizhou City.

## Author contributions

XX: Writing – review & editing, Writing – original draft, Methodology, Conceptualization. HY: Writing – review & editing, Methodology, Data curation. LH: Writing – review & editing, Visualization, Validation. WH: Writing – original draft, Supervision, Funding acquisition, Conceptualization. WC: Writing – review & editing, Resources, Project administration.
